# Supplementary material for: The Importance of Porins and β-Lactamase in Outer Membrane Vesicles on the Hydrolysis of β-Lactam Antibiotics
Source: Int J Mol Sci. 2020 Apr 17;21(8):2822. doi: 10.3390/ijms21082822 (PMC7215730; doi:10.3390/ijms21082822)
Supplement: Supplementary file 1 [file ijms-21-02822-s001.pdf]

Supplementary Materials

Table S1. Oligonucleotide primers.

The parts which serve as PCR primers for amplification of the cassette are underlined.

Description	Primer	Oligonucleotide sequence (5` to 3`)	Fragment size (bp)
Fragment 1	pRed N part SacI-F	ATGAGCTCGCCATCAGCTCAACCTGTTGAT	469
	pRed N part Cm^{R-}R	GATTTTTTTCTCCATACACGGTGCCTGACTGCGT	
Fragment 2	pRed N Cm^R connected-F	GTCAGGCACCGTGTATGGAGAAAAAAATCACTGG	688
	Cm^R pRed C-R	CAACGCGGGGAGGCTTACGCCCCGCCCTGCCAC	
Fragment 3	Cm^R pRed C connected-F	GCGGGGCGTAAGCCTCCCCGCGTTGCGTCG	253
	pRed C EcoRV-R	ATATGATATCATTCCTCCTGATCTCGAACCC	
Homology arms for blc1	FRT blc1 (upper oligo)	CTGGGTGTGGCATTGATTAACACAGCAGATAATTC GCAAATACTTTATCGAATTAACCCTCACTAAAGGGCG	-
	FRT blc1 (lower oligo)	TTAACGTCGGCTCGGTACGGTCGAGACGGAACGT TTCGTCTCCCAGCTGTTAATACGACTCACTATAGGGCTC	
Homology arms for ompC	FRT ompC (upper oligo)	CTGACAACAAAGATGTAGATGGCGACCAGACCTAC ATGCGTCTTGGCTTCAATTAACCCTCACTAAAGGGCGG	-
	FRT ompC (lower oligo)	TACTGAACAGCAAAGTTCAGGCCGTCAACCAGACC GAAGAAGTCAGTGTTTAATACGACTCACTATAGGGCTCG	
Homology arms for ompF	FRT ompF (upper oligo)	CGTAACTACGGTGTGGTTTATGATGCACTGGGTTAC ACCGATATGCTGCCAATTAACCCTCACTAAAGGGCG	-
	FRT ompF (lower oligo)	CAGGTAGATGTTGTTCGCGTCATACTTCAGACCAGT AGCCCACTGTTCAGTAATACGACTCACTATAGGGCTC	

Table S2. The MIC of β-lactam antibiotics against the β-lactam-susceptible *Escherichia coli* (RC85).

Antibiotics	MIC (μg/mL)[a]
	RC85
Ampicillin	8
Cefotaxime	≤1/4
Cefoperazone	≤1/4
Amoxicillin	4
Cefazolin	1
Cefalexin	4

[a]MIC indicates minimum inhibitory concentration

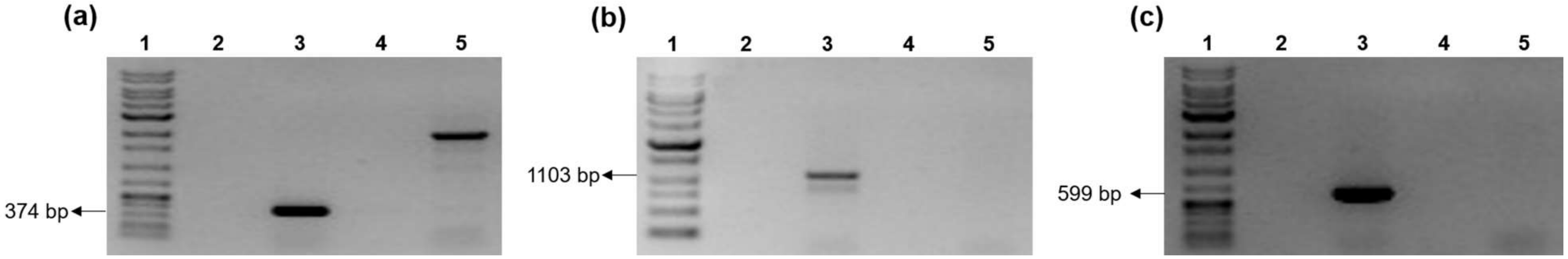

Figure S1. Gene deletion profile analysis in blc1 (a), ompC (b), and ompF (c) using colony PCR followed by 1% agarose gel electrophoresis.

Lane 1: molecular weight marker; lane 2: DNA blank; 3: RC85^{+}; 4: DNA blank; 5: mutant strain.

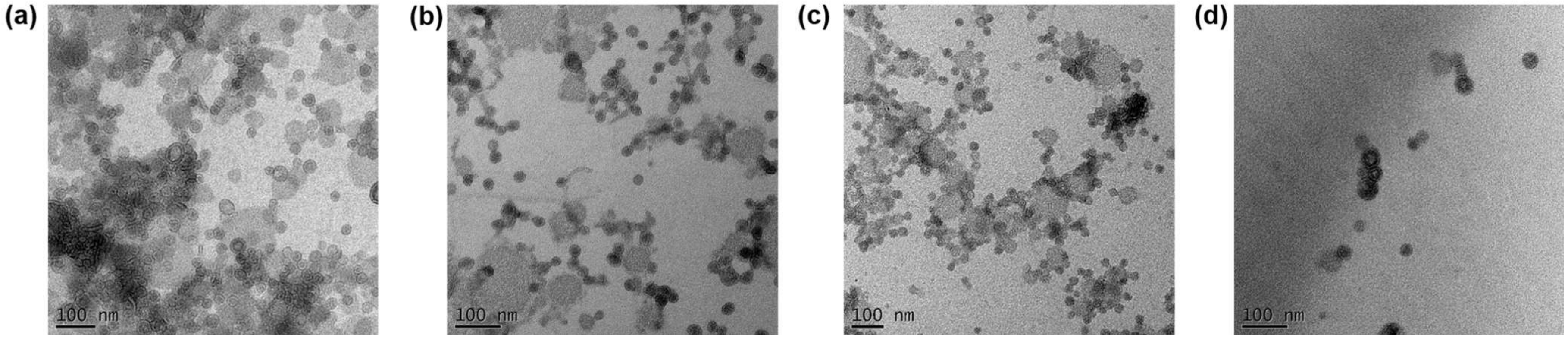

Figure S2. TEM images of OMVs released from RC85$^+$ (a), Δblc1 (b), ΔompC (c), and ΔompF (d) cells. Bar = 100 nm.

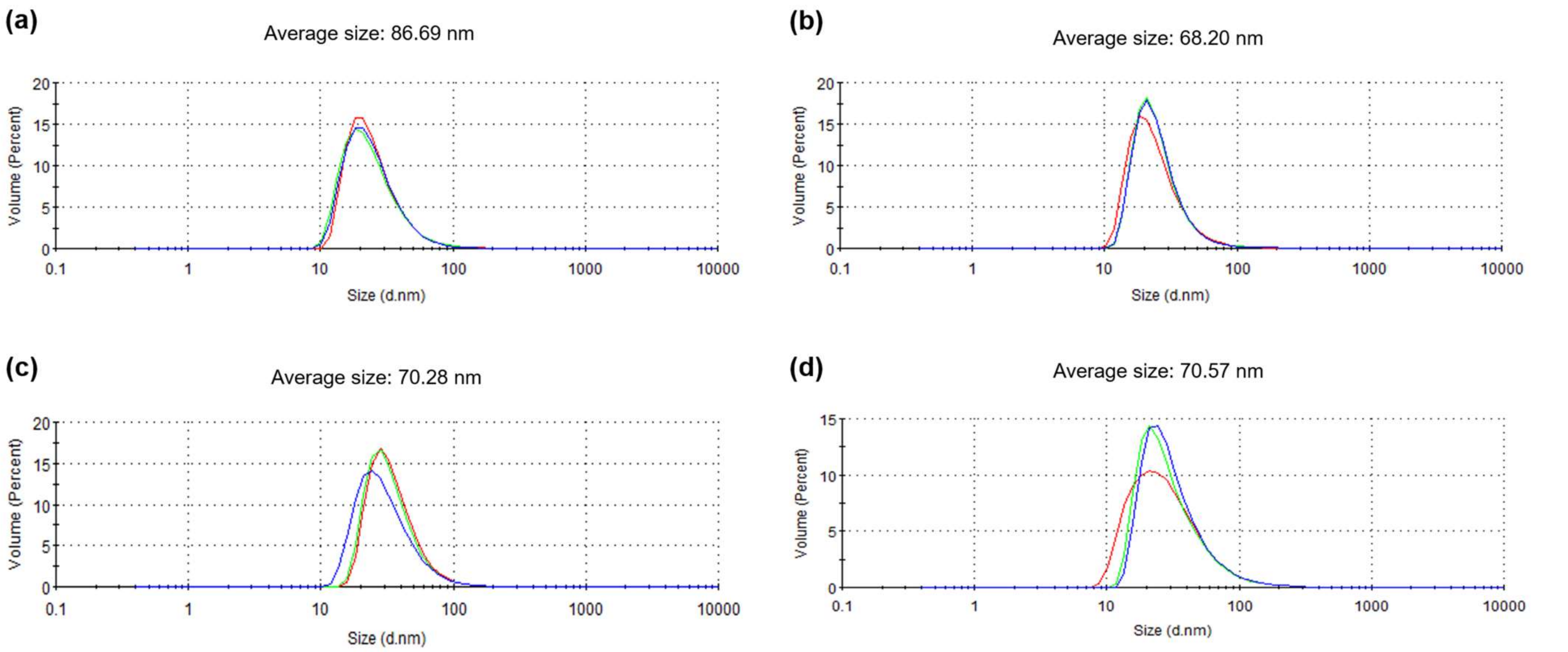

Figure S3. The size distribution OMVs derived from RC85$^+$ (a), Δblc1 (b), ΔompC (c), and ΔompF (d) cells, as assessed by Zeta-sizer. Three independent measurements were performed.
